# “Sweeter than a rose”, at least to *Triatoma phyllosoma* complex males (Triatominae: Reduviidae)

**DOI:** 10.1186/s13071-018-2677-z

**Published:** 2018-02-17

**Authors:** Irving J. May-Concha, Leopoldo C. Cruz-López, Julio C. Rojas, Janine M. Ramsey

**Affiliations:** 1 Centro Regional de Investigación en Salud Pública, Instituto Nacional de Salud Pública (CRISP-INSP), Tapachula, Chiapas Mexico; 2 Laboratorio de Investigación en Triatominos, Centro de Referencia de Vectores, Ministerio de Salud de la Nación (CeReVe), Hospital Colonia, Pabellón Rawson calle s, /n Córdoba, Argentina; 3 Grupo de Ecología y Manejo de Artrópodos, El Colegio de la Frontera Sur (ECOSUR), Tapachula, Chiapas Mexico

**Keywords:** *Phyllosoma* complex, Exocrine glands, Volatile compounds, Chemotaxonomy

## Abstract

**Background:**

The *Triatoma phyllosoma* complex of *Trypanosoma cruzi* vectors (Triatominae: Reduviidae) is distributed in both Neotropical and Nearctic bioregions of Mexico.

**Methods:**

Volatile organic compounds emitted by disturbed *Triatoma longipennis*, *Triatoma pallidipennis* and *Triatoma phyllosoma*, and from their Brindley’s and metasternal glands, were identified using solid-phase microextraction coupled with gas chromatography-mass spectrometry.

**Results:**

Disturbed bugs and the metasternal glands from *T*. *phyllosoma* released or had significantly fewer compounds than *T. longipennis* and *T. pallidipennis*. Isobutyric acid was the most abundant compound secreted by disturbed bugs of the three species, while Brindley’s glands of all species produced another four compounds: propanoic acid, isobutyric acid, pentyl butanoate, and 2-methyl hexanoic acid. Two novel compounds, both rose oxide isomers, were produced in MGs and released only by disturbed females of all three species, making this the first report in Triatominae of these monoterpenes. The principal compound in MGs of both sexes of *T. longipennis* and *T. phyllosoma* was 3-methyl-2-hexanone, while *cis-*rose oxide was the principal compound in *T. pallidipennis* females. The major components in male effluvia of *T. pallidipennis* were 2-decanol and 3-methyl-2-hexanone.

**Conclusion:**

Discriminant analysis of volatile organic compounds was significant, separating the three species and was consistent with morphological and genetic evidence for species distinctions within the complex.

## Background

Insects release a wide range of volatile organic compounds (VOCs) into the environment, which may have communicative functions acting as semiochemicals, while others may be metabolic by-products, albeit precursors, for evolving purposeful communication [1]. VOCs are produced in specialized cells, exocrine glands, or by associated microorganisms [2, 3], and may differ in chirality or ratio in closely related insect species [4]. Pheromone composition may vary within populations of the same species, but they may also be associated with species-specific recognition [5]. Nonetheless, pheromone compounds used by closely related species have structural similarities, suggesting common biosynthetic pathways [5]. As a result, VOC profiles can be used as characters for phylogenetic inferences [6, 7].

**Fig. 1:**
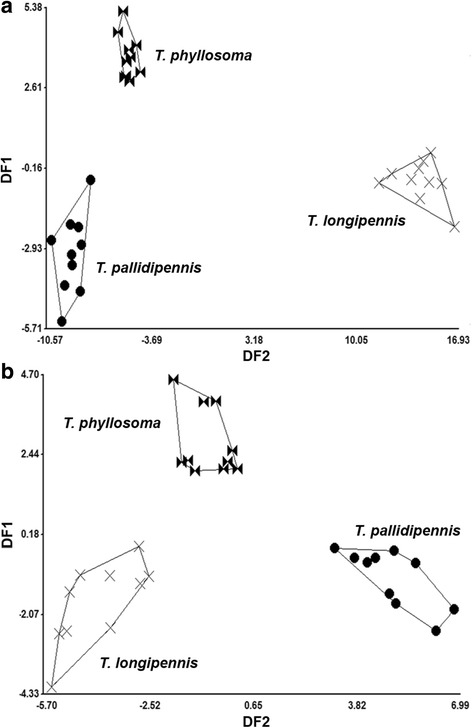
Discriminant analysis of volatiles released by disturbed adults of species of the *phyllosoma* complex. **a** Females. **b** Males. Each point represents one specimen on the canonical axis. Polygons enclose specimens of each species. DF1 and DF2 represent the discriminant function 1 and 2, respectively

**Fig. 2:**
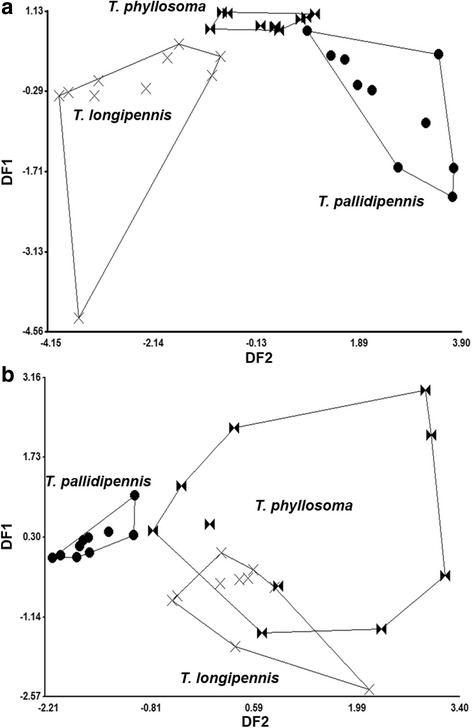
Discriminant analysis of compounds identified in GBs in species of the *phyllosoma* complex. **a** Females. **b** Males. Each point represents one specimen on the canonical axis. Polygons enclose specimens of each species. DF1 and DF2 represent the discriminant function 1 and 2, respectively

**Fig. 3:**
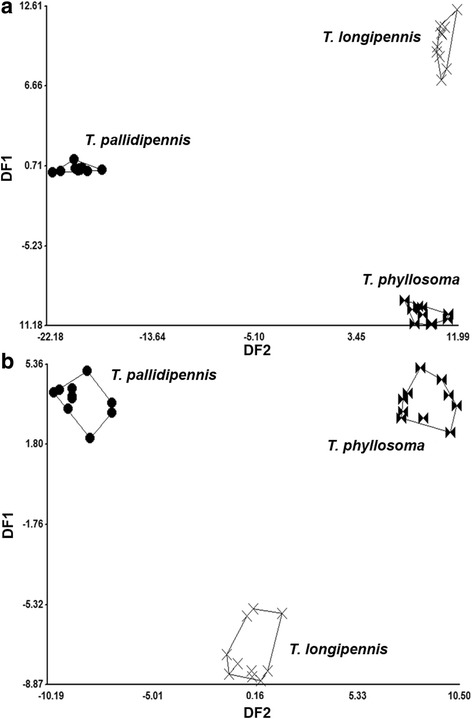
Discriminant analysis of compounds identified in MGs in species of the *phyllosoma* complex. **a** Females. **b** Males. Each point represents one specimen on the canonical axis. Polygons enclose specimens of each species. DF1 and DF2 represent the discriminant function 1 and 2, respectively

**Table 1:** Relative amount (in %, mean ± SE) of volatile compounds collected from the headspace of disturbed females and males of the three *phyllosoma* complex species

		*Triatoma longipennis*		*Triatoma pallidipennis*		*Triatoma phyllosoma*	
No.	Compound/ Sex	**♀**	**♂**	**♀**	**♂**	**♀**	**♂**
1	3-Methyl-2-pentanone	0.37 ± 0.04 (7)	t	0.51 ± 0.08 (6)	0.32 ± 0.04 (7)	0.05 ± 0.02 (9)	0.11 ± 0.03 (8)
2	3-Methyl-2-hexanone	2.77 ± 0.31 (10)	0.89 ± 0.16 (8)	nd	nd	0.77 ± 0.30 (9)	3.89 ± 0.97 (10)
3	4-Methyl-2-heptanol	0.11 ± 0.01 (8)	t	nd	nd	nd	nd
4	3,5-Dimethyl-2-hexanone	0.56 ± 0.03 (7)	t	nd	nd	nd	nd
5	2-Methyl-3-pentanol	0.4 ± 0.04 (8)	t	0.03 ± 0.01 (8)	1.47 ± 0.34 (10)	nd	nd
6	3,5-Dimethyl-2-hexanone isomer^a^	0.04 ± 0.00 (6)	t	nd	nd	nd	nd
7	6-Methyl-2-heptanol	0.37 ± 0.05 (7)	0.33 ± 0.04 (6)	nd	nd	nd	nd
8	2-Hexanol	0.05 ± 0.01 (8)	t	0.31 ± 0.07 (7)	1.52 ± 0.42 (10)	nd	nd
9	1-Octen-3-one	0.97 ± 0.21 (7)	0.31 ± 0.03 (8)	0.64 ± 0.09 (9)	0.56 ± 0.14 (10)	nd	nd
10	3-Methyl-2-hexanol	0.38 ± 0.04 (8)	0.32 ± 0.04 (6)	0.84 ± 0.22 (7)	0.36 ± 0.08 (10)	nd	nd
11	3-Methyl-2-hexanol isomer^a^	t	0.11 ± 0.02 (6)	nd	nd	nd	nd
12	*cis*-Rose oxide	9.82 ± 1.61 (10)	nd	7.53 ± 1.17 (10)	nd	8.81 ± 4.07 (10)	nd
13	*trans*-Rose oxide	5.63 ± 0.83 (10)	nd	5.62 ± 2.06 (10)	nd	2.43 ± 0.53 (10)	nd
14	2-Nonanol	t	0.69 ± 0.12 (7)	nd	nd	nd	nd
15	1-Octen-3-ol	1.73 ± 0.23 (6)	0.11 ± 0.02 (7)	0.16 ± 0.02 (8)	0.56 ± 0.15 (5)	nd	nd
16	3,5-Dimethyl-1-hexene	t	t	nd	nd	nd	nd
17	Decanal	0.86 ± 0.16 (7)	t	2.36 ± 0.5 (7)	3.04 ± 0.69 (8)	nd	nd
18	Propanoic acid	nd	nd	0.76 ± 0.15 (10)	0.38 ± 0.07 (10)	0.33 ± 0.10 (10)	0.41 ± 0.09 (10)
19	Isobutiryc acid	75.78 ± 3.16 (10)	96.77 ± 0.43 (10)	77.24 ± 5.11 (10)	90.34 ± 2.11 (10)	87.23 ± 4.83 (10)	94.73 ± 1.21 (10)
20	Pentyl butanoate	nd	nd	0.51 ± 0.17 (8)	1.1 ± 0.25 (10)	0.04 ± 0.01 (10)	0.37 ± 0.10 (9)
21	2-Methyl hexanoic acid	nd	nd	3.49 ± 0.94 (8)	0.35 ± 0.07 (10)	0.34 ± 0.09 (10)	0.49 ± 0.10 (10)

^a^Compound that was not identified by comparison to pure standards

*Note*: Numbers in parentheses indicate the detection frequency for each compound (*n* = 10 samples)

*Abbreviations*: *t*, traces (< 0.1% abundance); *nd*, not detected

**Table 2:** Relative amount (%, mean ± SE) of volatile compounds in the effluvia of Brindley’s glands of females and males of the three *phyllosoma* complex species

		*Triatoma longipennis*		*Triatoma pallidipennis*		*Triatoma phyllosoma*	
No	Compounds/ sex	**♀**	**♂**	**♀**	**♂**	**♀**	**♂**
1	Propanoic acid	4.11 ± 0.69 (10)	3.69 ± 0.45 (10)	0.67 ± 0.11 (10)	2.81 ± 0.45 (10)	0.91 ± 0.16 (10)	5.82 ± 1.10 (10)
2	Isobutyric acid	90.35 ± 2.46 (10)	92.56 ± 0.63 (10)	91.05 ± 1.94 (10)	96.31 ± 0.53 (10)	98.15 ± 0.19 (10)	88.01 ± 1.67 (10)
3	Pentyl butanoate	0.56 ± 0.12 (10)	0.72 ± 0.08 (10)	0.63 ± 0.24 (10)	0.11 ± 0.01 (10)	0.25 ± 0.03 (10)	0.59 ± 0.14 (10
4	2-Methyl hexanoic acid	4.98 ± 1.74 (10)	3.03 ± 0.27 (10)	7.65 ± 1.69 (10)	0.78 ± 0.11 (10)	0.68 ± 0.12 (10)	5.58 ± 1.57 (10)

*Note*: Numbers in parentheses indicate the detection frequency for each compound (*n* = 10 samples)

**Table 3:** Relative amount (%, mean ± SE) of volatile compounds in the effluvia of Metasternal glands of females and males of the three *phyllosoma* complex species

		*Triatoma longipennis*		*Triatoma pallidipennis*		*Triatoma phyllosoma*	
No	Compounds/ sex	**♀**	**♂**	**♀**	**♂**	**♀**	**♂**
1	2-Pentanone	0.19 ± 0.02 (7)	0.29 ± 0.03 (8)	0.1 ± 0.02 (6)	1.33 ± 0.12 (7)	0.15 ± 0.02 (6)	t
2	3-Methyl-2-pentanone	5.58 ± 0.41 (8)	9.94 ± 0.90 (7)	3.11 ± 0.47 (8)	12.56 ± 0.86 (8)	8.63 ± 1.11 (8)	2.42 ± 0.42 (7)
3	2-Butanol	nd	nd	0.83 ± 0.14 (6)	1.76 ± 0.12 (6)	nd	nd
4	2-Methyl-3-buten-2-ol	nd	nd	0.49 ± 0.06 (8)	1.19 ± 0.12 (8)	nd	nd
5	3-Methyl-2-hexanone	34.65 ± 1.72 (10)	45.48 ± 3.07 (10)	8.93 ± 0.94 (10)	18.52 ± 2.34 (9)	73.99 ± 0.08 (9)	90.84 ± 1.12 (9)
6	4-Methyl-2-heptanol	1.1 ± 0.06 (7)	1.44 ± 0.09 (8)	1.35 ± 0.15 (6)	3.1 ± 0.56 (7)	nd	nd
7	3,5-Dimethyl-2-hexanone	3.57 ± 0.30 (8)	3.14 ± 0.26 (7)	0.44 ± 0.06 (7)	2.69 ± 0.23 (8)	t	1.2 ± 0.13 (7)
8	2-Methyl-3-pentanol	4.44 ± 0.23 (9)	1.75 ± 0.13 (6)	1.03 ± 0.08 (8)	4.82 ± 0.58 (7)	nd	nd
9	4-Methyl-2-pentanol	0.15 ± 0.01 (8)	0.17 ± 0.02 (5)	nd	nd	nd	nd
10	3,5-Dimethyl-2-hexanone isomer^a^	0.12 ± 0.01 (8)	0.17 ± 0.02 (8)	nd	nd	0.2 ± 0.04 (8)	0.84 ± 0.13 (7)
11	6-Methyl-2-heptanol	13.42 ± 1.01 (6)	17.57 ± 1.18 (7)	2.95 ± 0.16 (6)	1.15 ± 0.14 (5)	0.4 ± 0.07 (7)	2.91 ± 0.40 (8)
12	2-Methyl-1-butanol	nd	nd	1.11 ± 0.20 (7)	3.6 ± 0.51 (6)	t	t
13	5-Methyl-2-heptanol	0.1 ± 0.01 (7)	0.15 ± 0.02 (6)	nd	nd	nd	nd
14	2-Hexanol	0.16 ± 0.01 (9)	0.49 ± 0.04 (7)	0.4 ± 0.03 (8)	3.58 ± 0.45 (7)	nd	nd
15	2-Heptanol	nd	nd	nd	nd	0.22 ± 0.04 (6)	t
16	1-Octen-3-one	1.8 ± 0.19 (10)	1.48 ± 0.12 (9)	0.61 ± 0.05 (9)	1.55 ± 0.20 (9)	0.28 ± 0.03 (8)	t
17	3-Methyl-2-hexanol	0.37 ± 0.05 (10)	7.87 ± 1.40 (9)	4.79 ± 0.05 (9)	13.35 ± 1.45 (8)	1.04 ± 0.17 (9)	0.9 ± 0.18 (8)
18	3-Methyl-2-hexanol isomer^a^	0.18 ± 0.01 (6)	0.41 ± 0.05 (8)	0.68 ± 0.89 (7)	1.74 ± 0.17 (6)	0.1 ± 0.01 (7)	t
19	2-Decanol	nd	nd	3.67 ± 0.04 (6)	19.65 ± 1.77 (7)	0.25 ± 0.06 (5)	0.2 ± 0.04 (8)
20	*cis*-Rose oxide	10.87 ± 0.41 (10)	nd	44.42 ± 0.53 (8)	nd	11.25 ± 0.83 (8)	nd
21	*trans*-Rose oxide	3.84 ± 0.41 (10)	nd	17.66 ± 2.58 (9)	nd	2.43 ± 0.43 (9)	nd
22	2-Nonanol	1.24 ± 0.13 (7)	6.69 ± 0.1 (6)	nd	nd	nd	nd
23	1-Octen-3-ol	16.27 ± 1.79 (9)	6.69 ± 0.63 (7)	3.03 ± 0.10 (7)	3.49 ± 0.41 (8)	0.12 ± 0.01 (8)	0.11 ± 0.03 (8)
24	3,5-Dimethyl-1-hexene	0.18 ± 0.01 (8)	0.84 ± 0.06 (9)	nd	nd	0.19 ± 0.02 (7)	0.14 ± 0.03 (7)
25	Decanal	1.78 ± 0.11 (10)	1.44 ± 0.14 (9)	4.41 ± 0.50 (8)	5.92 ± 0.48 (9)	1.15 ± 0.12 (8)	t

^a^Compounds not identified by comparison to pure standards

*Note*: Numbers in parentheses indicate the detection frequency for each compound (*n* = 10 samples)

*Abbreviations*: t, traces (< 0.1% abundance); nd, not detected

Triatomine bugs have two principal exocrine glands: the Brindley’s (BGs) and the metasternal (MGs) glands [8]. Previous studies demonstrated that these glands in some triatomines produce and release VOCs for alarm and sexual behaviour [9–12]. BGs of most triatomines primarily release isobutyric acid, a highly conserved compound in both North and South American sub-clades of the Triatomini [13]. In contrast, MGs produce multiple ketones and alcohols, most of which are species-specific [10–12, 14, 15]. A recent study comparing exocrine volatiles among the three primary *dimidiata* complex haplogroups demonstrated subtle but specific differences in production and secretion of VOCs between sister species [12].

One of the largest and epidemiologically important triatomine species complexes in North America, is the *Triatoma phyllosoma* complex [16–19]. It only occurs in Mexico, broadly distributed from the northeastern Nearctic through to the northern Neotropical bioregion of Mexico, west of the Tehuantepec Isthmus/Chimalapas forest. Species of the *phyllosoma* complex are principal vectors of *Trypanosoma cruzi* in sylvatic and synanthropic habitats in Mexico, due to their broad opportunism for biotic interactions, and their amplification in modified habitats [20–24]. The most widely accepted taxonomic revision of the Triatominae returned all sub-species of the *phyllosoma* complex (described to that date) to species-level classification based on classical morphology (i.e. *T. phyllosoma*, *T. picturata*, *T. pallidipennis*, *T. longipennis* and *T. mazzottii*) [16]. Three subsequently described species were added (*T. brailovskyi*, *T. bolivari*, *T. bassolsae*), and three further species were included in the complex based on molecular evidence (*T. gerstaeckeri*, *T. recurva* and *T. mexicana*) [18, 19, 25]. In many regions, two or more species of the complex are sympatric, and although there is clear reproductive exclusion (after backcrossing), successful first- and second-generation reproduction between species has been reported ([26, 27], J. Ramsey, personal communication).

Given significant differences in VOC concentrations and aggregation or alarm behaviour among species of the closely related *dimidiata* complex, we hypothesized that VOC profiles would be similar in the *phyllosoma* complex species. Geographically, *T. pallidipennis* and *T. longipennis* are sympatric across part of their distributions, while *T*. *phyllosoma* is allopatric to both, which could have an impact on production or expression of conserved VOCs. Additionally, given novel VOCs identified in all *dimidiata* complex haplogroups, we also hypothesized that the phylogenetically close *phyllosoma* complex would share these or similar novel compounds. Alarm pheromones may give rise to a plethora of secondary compounds and behaviours, and hence by focusing on understanding disturbed behaviour, complex interactions which specifically affect speciation or population dynamics, such as improved vector fitness in modified habitats, can be analyzed. VOCs emitted by disturbed adults of *T. pallidipennis*, *T. longipennis* and *T. phyllosoma*, as well as those produced by their BGs and MGs, are therefore important not only to provide complementary genetic and ethological characters to understand speciation drivers and phylogeography, but their understanding is essential to develop effective and sustainable control tools effective for specific eco-demographic patterns related to land use and vector exposure potential [28].

## Methods

### Triatomine populations tested

*Triatoma pallidipennis*, *T. longipennis* and *T. phyllosoma* colonies were established at the Centro Regional de Investigación en Salud Pública (CRISP/INSP) from collection sites where species are not sympatric. *Triatoma pallidipennis* was colonized using individuals collected across the state of Morelos, while the *T. longipennis* colony was derived from specimens from Zacatecas state, the only region where it is not sympatric with another species of the *phyllosoma* complex. *Triatoma phyllosoma* was collected from Pacific coast collection sites surrounding the Tehuantepec Isthmus (Salina Cruz, Ixtepec, Mexico). All colonies were bred to no more than four generations before new individuals from original sites were introduced to breeding pairs. All were maintained at 27 ± 2 °C, 70 ± 5% RH, and a photoperiod of 12:12 (Light:Dark). New Zealand White (NZW) rabbits were used as a blood meal source for all bugs, and these latter were fed eight days before sampling their volatiles. Fifth-stage nymphs were maintained separately and sexed once emerged, in single-sex cohorts, to ensure that adult insects used in the experiments were virgins. Experiments were performed with adults between 15 and 30 days after emergence. Each insect was sampled only once.

### Volatile sampling and chemical analyses

Volatiles emitted by triatomines, and those produced by MGs and BGs were sampled using solid phase microextraction devices (SPME) fitted with fibers coated with 65 μm polydimethylsiloxane-divinylbenzene (PDMS-DVB; Supelco, Belfonte, PA, USA). Previous studies have shown that this fiber efficiently samples the volatile compounds emitted by triatomine bugs [11, 12]. The fiber was exposed to the insect headspace for 60 min, and all samples were maintained at the same temperature and relative humidity (25 ± 2 °C and 65 ± 10% RH). In all experiments, control using the same conditions was performed before each test, using an empty flask. After the sampling period, the fiber was withdrawn and inserted into the injector of a gas chromatograph-mass spectrometer (GC-MS). The samples were desorbed for 1 min in the GC injector for analysis.

GC-MS analyses were performed with a GC Varian model CP-3800 equipped with a polar CP-wax 57CB capillary column (25 m by 0.32 mm, and 0.20 μm coat thickness) coupled with a Varian Saturn 2200 mass spectrometer (Varian, Palo Alto, CA, USA). The oven temperature was programmed for 40 °C (1 min hold), then 10 °C min^-1^ to 75 °C (0 min), then 15 °C min^1^ to 200 °C, and held for 15 min. The splitless mode was used for the injector with the inlet temperature set at 250 °C. Helium was used as a carrier gas at 1.0 ml min^-1^. Ionization was by electron impact at 70 eV. The compounds identified had relative abundance ≥ 0.1% and were tentatively identified by matching the mass spectra of GC peaks with those in the MS library (NIST 2002). The identities of the compounds were confirmed by comparing retention times with mass spectra of synthetic standards. The relative abundance of a particular compound was calculated as the proportion of its area to all GC peak areas combined.

Standard compounds for most of those identified in the headspace of disturbed bugs and exocrine glands were obtained at 98–99.5% purity from commercial sources (Sigma-Aldrich, Toluca, Mexico). 3-methyl-2-hexanone and 3-methyl-2-pentanone were donated by Dr. William F. Wood (Chemistry Department, Humboldt State University, Arcata, CA, USA); 3,5-dimethyl-2-hexanone was donated by Dr. Joselyn G. Millar (Department of Entomology, University of California, Riverside, CA, USA). 3-methyl-2-hexanol was prepared from 3- methyl-2-hexanone by reduction with sodium borohydride in methanol [29]. 1-octen-3-one was prepared by sodium hypochlorite oxidation of 1-octen-3-ol [29].

### Volatiles released by disturbed adults

Volatiles released by disturbed and undisturbed adults of the three species were identified from replicates using either three females/group, or three males/group. Bugs were gently introduced into a 50 ml borosilicate glass Erlenmeyer flask and the mouth of the flask covered with aluminum foil and sealed with masking tape. The experimental bugs were vigorously shaken for 30 s, while control bugs were not. An SPME fiber was exposed immediately to the headspace through a pin-size hole in the top of the aluminum foil. After 60 min, the fiber was withdrawn and inserted into the GC-MS injector. Ten replicates each of shaken or unshaken females or males were performed.

### Volatiles produced by BGs and MGs

Volatile compounds from female and male BGs and MGs were identified in all *T*. *phyllosoma* complex species. Bugs were placed in a freezer at -20 °C for 5 min to avoid spontaneous emptying of the glands during processing, and ten pairs of MGs and BGs from both females and males were dissected separately under sterile water using a binocular microscope. Glands were placed in pairs into a 2 ml glass conical vial and the mouth of the vial covered with aluminum foil sealed with masking tape. The glands were crushed using a thin wire, which was introduced into the vial through a pin-size hole in the aluminum foil, an SPME fiber was then exposed to the headspace for 60 min after which the fiber was withdrawn and inserted into the GC-MS injector. Ten replicates of each sex, species, and type of gland were sampled.

### Statistical analyses

Differences between VOCs released by disturbed adults and by exocrine glands among species were analyzed using univariate and multivariate methods. Means and standard deviations of abundance were calculated for each chemical compound. Since the variables were heteroscedastic, the Kruskall-Wallis non-parametric test was used for univariate analyses. The software PAD version 60 was used to compare differentially among the three species, or according to sex (10 males, 10 females for each species), for the compounds produced by MGs and BGs, or released by disturbed bugs. PAD uses a classical discriminant analysis to estimate non-parametric statistical significance (Wilks and Mahalanobis distance values) using a permutation test (1000 permutation) and uses crosschecked classification tests to validate the re-classification of discriminant analysis [30].

## Results

### Volatiles released by disturbed adults

Disturbed females from *T. longipennis*, *T. pallidipennis* and *T. phyllosoma* released 18, 13 and 8 compounds, respectively, while disturbed males emitted 16, 11 and 6 compounds, respectively (Table 1). The compounds 3-methyl-2-pentanone and isobutyric acid were common to both sexes of all three species, the latter accounting for 76–97% of the volatiles released by disturbed bugs. The compounds *cis*-rose oxide and *trans*-rose oxide were emitted only by females of all three species. Compounds 3, 4, 6, 7, 11, 14 and 16 were exclusively emitted by *T. longipennis*, while compounds 18, 20 and 21 were found in the headspace only of *T. pallidipennis* and *T. phyllosoma* (Table 1). *Triatoma phyllosoma* disturbed bugs released significantly fewer compounds as compared to those released by *T. pallidipennis* and *T. longipennis*.

There were significant differences in the relative amounts of VOCs emitted by disturbed bugs among the three species (*H* = 42.75, *df* = 5, *P* = 0.01). Disturbed females from the three species emitted significantly different amounts of 21 compounds (*H* = 19.35, *df* = 2, *P* = 0.03). However, both sexes of *T. longipennis* released similar amounts of 6-methyl-2-heptanol (*H* = 0.46, *df* = 1, *P* = 0.49) and 3-methyl-2-hexanol (*H* = 1.75, *df* = 1, *P* = 0.18), while there were no differences in the relative amounts of 3-methyl-2-pentanone (*H* = 2.64, *df* = 1, *P* = 0.10), 1-octen-3-one (*H* = 0.69, *df* = 1, *P* = 0.40), and decanal (*H* = 1.46, *df* = 1, *P* = 0.22) emitted by male and female *T. pallidipennis*. There was no difference in the relative amount of 2-methyl hexanoic acid (*H* = 1.56, *df* = 1, *P* = 0.21) released by either sex of *T. phyllosoma*, and there was no significant difference in 3-methyl-2-pentanone (*H* = 19.38, *df* = 2, *P* > 0.05), 1-octen-3-one (*H* = 19.46, *df* = 2, *P* > 0.05), 3-methyl-2-hexanol (*H* = 23.08, *df* = 2, *P* > 0.05), and isobutyric acid (*H* = 11.82, *df* = 2, *P* > 0.05) concentrations between *T. longipennis* and *T. pallidipennis*. Female *T*. *phyllosoma* emitted significantly higher amounts of isobutyric acid in comparison to *T. pallidipennis* and *T. longipennis* females (*H* = 11.82, *df* = 2, *P* = 0.002), while *T. longipennis* males released significantly higher amounts of isobutyric acid than males of the other two species (*H* = 12.43, *df* = 2, *P* = 0.002). Disturbed males secreted significantly different quantities of 19/21 compounds (*H* = 23.28, *df* = 5, *P* = 0.001), with *T. phyllosoma* males releasing fewer compounds compared to *T. pallidipennis* and *T. longipennis.* There was no quantitative difference of pentyl butanoate (*H* = 22.43, *df* = 2, *P* > 0.05) or 2-methyl hexanoic acid (*H* = 19.83, *df* = 2, *P* > 0.05) between *T. pallidipennis* and *T. phyllosoma* males.

### Volatiles produced in GBs

BGs of all three *T*. *phyllosoma* complex species emitted four compounds: propanoic acid, isobutyric acid, pentyl butanoate, and 2-methyl hexanoic acid (Table 2). The major compound from BGs of all three species was isobutyric acid, which was also the major compound in the effluvia of disturbed bugs (Table 1). There were interspecific differences of the four BG compounds among females of the three species (*H* = 10.54, *df* = 2, *P* = 0.005). BGs of *T. phyllosoma* females released the highest amount of isobutyric acid (*H* = 19.40, *df* = 2, *P* = 0.001), while there were no differences in the relative amounts of isobutyric acid (*H* = 19.09, *df* = 2, *P* > 0.05), pentyl butanoate (*H* = 10.54, *df* = 2, *P* > 0.05), or 2-methyl hexanoic acid (*H* = 22.00, *df* = 2, *P* > 0.05) between females of *T. longipennis* and *T. pallidipennis*. Similarly, there were significant differences for all four BG compounds in males between species (*H* = 17.11, *df* = 2, *P* = 0.001). *Triatoma pallidipennis* males released the highest amount of isobutyric acid, as compared to *T. longipennis* and *T. phyllosoma* (*H* = 18.40, *df* = 2, *P* = 0.0001), whereas there was no difference in the relative amounts of pentyl butanoate (*H* = 16.11, *df* = 2, *P* > 0.05) and 2-methyl hexanoic (*H* = 20.84, *df* = 2, *P* > 0.05) acid between these latter two species.

BGs from *T. longipennis* males had a higher amount of pentyl butanoate (*H* = 6.22, *df* = 1, *P* = 0.01) than females. BGs from *T. pallidipennis* females had a higher amount of 2-methyl hexanoic acid (*H* = 14.29, *df* = 1, *P* = 0.0002) than conspecific males, while in contrast, there were higher amounts of propanoic (*H* = 13.72, *df* = 2, *P* = 0.0002) and isobutyric acid (*H* = 7.82, *df* = 2, *P* = 0.005) in males as compared to females of the same species. BGs from *T. phyllosoma* females contained more isobutyric acid (*H* = 14.29, *df* = 2, *P* = 0.0002) than males, while BGs from the same species’ males contained significantly higher amounts of propanoic (*H* = 14.29, *df* = 2, *P* = 0.0002) and 2-methyl hexanoic acid (*H* = 14.29, *df* = 2, *P* = 0.0002) in comparison to females.

### Volatiles contained in MGs

MGs from *T. longipennis*, *T. pallidipennis*, and *T. phyllosoma* females contained 20, 19 and 17 compounds, respectively, while MGs from males contained 18, 17 and 15 compounds, respectively (Table 3). Five compounds (2, 5, 11, 17 and 23) were present in more than trace quantities in both sexes of all three species. The principal MG compound in both sexes of *T. longipennis* and *T. phyllosoma* was 3-methyl-2-hexanone (compound 5). Although *cis-*rose oxide (compound 20) was the principal compound in *T. pallidipennis* females, the major components in effluvia of male MGs were 3-methyl-2-hexanone and 2-decanol. Compounds 3 and 4 were exclusively found in *T. pallidipennis*, while compounds 9, 13 and 22 were exclusively identified in *T. longipennis*. There was a significant difference in the relative amounts of 25 MG compounds between females of the three species (*H* = 15.68, *df* = 2, *P* = 0.0004). MGs contained, however, only 17 of the 21 volatile compounds emitted by disturbed bugs (Table 1). *Triatoma phyllosoma* released the highest amount of 3-methyl-2-hexanone of the three species (*H* = 25.81, *df* = 2, *P* = 0.0001), while *T. longipennis* and *T. pallidipennis* secreted similarly high amounts of 4-methyl-2-heptanol (*H* = 19.90, *df* = 2, *P* > 0.05). There were also significant differences for 23 (of 25) compounds identified in male MGs between the three species (*H* = 25.81, *df* = 2, *P* = 0.001). *Triatoma phyllosoma* male MGs had a significantly higher amount of 3-methyl-2-hexanone (*H* = 23.69, *df* = 2, *P* = 0.0001), while the amounts of 1-octen-3-one were similar, between *T. longipennis* and *T. pallidipennis* (*H* = 20.90, *df* = 2, *P* > 0.05).

Interestingly, compounds 20 (*cis*-rose oxide) and 21 (*trans*- rose oxide) were only identified in female MGs of the three species. There were no significant differences in the compounds 3-methyl-2-hexanone (*H* = 0.01, *df* = 1, *P* = 0.93), 4-methyl-2-heptanol (*H* = 0.37, *df* = 1, *P* = 0.54), and 6-methyl-2-heptanol (*H* = 0.37, *df* = 1, *P* = 0.52) between males and females of *T. longipennis*, whereas there was no significant difference for only 1-octen-3-ol (*H* = 2.17, *df* = 1, *P* = 0.14) between male and female *T. pallidipennis*. There were significant differences in the amounts of 3-methyl-2-pentanone (*H* = 13.71, *df* = 1, *P* = 0.0002), 3–5-dimethyl-2-hexanone (*H* = 14.29, *df* = 1, *P* = 0.0002), 1-octen-3-one (*H* = 13.17, *df* = 1, *P* = 0.0003), and decanal (*H* = 14.29, *df* = 1, *P* = 0.0002) between male and female *T. phyllosoma*.

### Intra-complex differences in disturbed, BG, and MG adult volatiles

The first function of the linear discriminant analysis of 21 compounds from disturbed females explained 84.9% of the total variation, and the second function explained 15.2%. The Mahalanobis distances for females were significantly different among the three species (*P* < 0.0001), with the discriminant plot indicating that *T. longipennis* is the most distant from both *T. pallidipennis* and *T. phyllosoma* (Fig. 1a). The Mahalanobis distance (D2) for the female group centroid indicated that *T. longipennis* is more distant from *T. pallidipennis* (D2 = 23.52), than from *T. phyllosoma* (D2 = 20.09), while *T. pallidipennis* was least distant from *T. phyllosoma* (D2 = 7.78) (Fig. 1a). The first discriminant function for disturbed males explained 74.4% of total variation, whereas the second function explained 25.6%, and the Mahalanobis distances for males indicated significant differences among the three species (*P* < 0.01). The discriminant plot of disturbed males indicated that *T. pallidipennis* is most distant from *T. longipennis* and *T. phyllosoma* (Fig. 1b). The Mahalanobis distance (D2) for the male group centroids, indicated that *T. pallidipennis* is more distant from *T. longipennis* (D2 = 9.07) than from *T. phyllosoma* (D2 = 6.83), whereas *T. phyllosoma* males were least distant to *T. longipennis* (D2 = 5.66) (Fig. 1b).

The first function of the linear discriminant analysis of the four female BG compounds explained 90.7% of total variation, whereas the second function explained 9.3%. The Mahalanobis distances for females among the three species differed significantly (*P* < 0.0001), and the discriminant plot separated *T. longipennis* from *T. pallidipennis,* and the former from *T. phyllosoma* (Fig. 2a). The Mahalanobis distance (D2) based on female group centroids was greater between *T. longipennis* and *T. pallidipennis* (D2 = 5.02) as compared to the former and *T. phyllosoma* (D2 = 2.99), while *T. pallidipennis* was closest to *T. phyllosoma* (D2 = 2.79) (Fig. 2a). The first linear discriminant function for male BGs explained 80.8% of total variation, whereas the second function explained 19.2%. The Mahalanobis distances for males were not significantly different between *T. longipennis* and *T. phyllosoma* (*P* > 0.01); the greatest distance for males was between *T. pallidipennis* and *T. longipennis,* as well as the former and *T. phyllosoma* (*P* < 0.0001). The Mahalanobis distance (D2) for male group centroids was greatest between *T. pallidipennis* and *T. phyllosoma* (D2 = 2.88), followed by the former to *T. longipennis* (D2 = 2.29), while *T. phyllosoma* males were closest to *T. longipennis* males (D2 = 1.60) (Fig. 2b).

Linear discriminant analysis of MGs between males and females included 25 compounds. The first discriminant function explained 90.3% of the total variation for females, whereas the second function for females explained 9.3%. The Mahalanobis distances for females were significantly different among the three species (*P* < 0.0001), with *T. pallidipennis* being the most distant from *T. longipennis* and *T. phyllosoma* (Fig. 3a). The Mahalanobis distance (D2) for the female group centroids, indicated that *T. pallidipennis* was more distant from *T. longipennis* (D2 = 32.00) than from *T. phyllosoma* (D2 = 31.09), while *T. phyllosoma* was closest to *T. longipennis* (D2 = 20.22) (Fig. 3a). The first discriminant function for male MGs explained 99.3% of total variation, whereas the second function explained 0.7%. The Mahalanobis distances for males were significantly different among the three species (*P* < 0.0001), with *T. phyllosoma* the most distant from both *T. longipennis* and *T. pallidipennis* (Fig. 3b). The Mahalanobis distance (D2) for the male group centroids indicated that *T. phyllosoma* is more distant from *T. pallidipennis* (D2 = 17.50) than from *T. longipennis* (D2 = 14.25), while *T. pallidipennis* was closest to *T. longipennis* (D2 = 14.19) (Fig. 3b).

## Discussion

There are qualitative and quantitative differences in VOCs emitted by disturbed bugs, and produced by exocrine glands, among the three *phyllosoma* complex species. The compound profiles differ between sexes of all three species; *T. phyllosoma* released significantly fewer VOCs than the other two northern and sympatric species. *Triatoma phyllosoma*, allopatric with *T. longipennis* and *T. pallidipennis* is confined to lower altitudes immediately east and south of the Zapotecan foothills west of the Tehuantepec Isthmus, in a geographically confined semi-arid region along the Pacific coast of Oaxaca in Mexico. This species is only sympatric with *T. dimidiata* haplogroup 2 in this region [19, 24].

Despite vast latitudinal and altitudinal differences among the distributions of the three species of the *phyllosoma* complex, and hence important host species turnover, all three species produced and secreted the rose oxide compounds [19]. This unique phenotype was independent of the biogeographical region (Neotropical and Nearctic) or allopatry, since it was conserved in all three species. It is interesting that both closely related *T*. *phyllosoma* and *T. dimidiata* complexes have independently (and recently) developed distinct unique specific VOC communication compounds. In both complexes, they are conserved through speciation, although species-level and gender-specific concentration, behaviour, or receptor differences occur between species [31].

The *phyllosoma* complex MGs contained, depending on the species, between 17 and 25 compounds each, nine of which were also identified from the *dimidiata* complex, which provides additional evidence for their close phylogenetic relationships [12, 25, 32]. Approximately 80% (17) of VOCs emitted by disturbed bugs were produced by MGs, although 10 additional MG compounds were not secreted by disturbed bugs. Some of these compounds have also been identified in the headspace of *T. dimidiata* mating pairs, one of which, 3-methyl-2-hexanone, was attractive for conspecific males [11]. 3-Methyl-2-hexanone has also been reported from MGs of *D. maximus* [33], a closely-related species, while 3-methyl-2-hexanol has been reported from *T. brasiliensis* [15, 34]. The compound 2-methyl-3-buten-2-ol is conserved at the tribal level, having been reported from *Rhodnius prolixus* of the Rhodniini [14].

Rose oxide compounds have been reported in plants such as *Rosa damascena* Mill [35], *Pelargonium graveolens* Ait [35], *Ribes nigrum* Linnaeus [36], *Hamanasu* sp. [37] and *Physalis peruviana* Linnaeus [38]. In insects, rose oxide isomers have been reported from the secretions of two coleopterans, for which they are thought to have a defensive function [39–41]. They have also been reported, although not for alarm, from *Melipona beecheii* [42]. In the Triatominae [33] and Cerambycidae [43], the paired MG is located in the thorax and has reservoirs with openings near the articulated coxa of the hind legs. The oxide isomers may, therefore in females of the *phyllosoma* complex, function during copula, similar to that observed in some cerambycids which emit defensive semiochemicals from MGs [39, 40]. Although the relationship between chirality and behaviour-modifying chemicals perceived through olfaction has been studied in insects [44], the impact of chirality on insect behaviour and for specific activities has not been analyzed. In triatomines, the two novel enantiomers, the *cis* and *trans* isomers of rose oxide, represent a paradigm for olfactory-guided behaviour. These female-specific compounds may act as sexual and alarm pheromones, or as early warning signals of predators, hypotheses currently being analyzed [41, 45].

One of the major BG components of the blend emitted by disturbed bugs, from all three species, was isobutyric acid. This compound has been reported as a major component from disturbed *T. infestans* [10, 46, 47], *R. prolixus* and *D. maximus* [48], the *dimidiata* complex [12], and other hematophagous arthropods such as ticks [49]. Isobutyric acid was the most abundant BG compound in all species of the *phyllosoma* complex, as in several other triatomine species [8, 12, 50, 51]. Some studies propose a role for isobutyric acid in alarm and defense [10, 12, 47, 52, 53], in addition to its involvement in sexual communication [11, 48, 54]. Pentyl butanoate has also been reported in bedbugs of the genus *Cimex*, while hexanoic acid has been reported in certain Diptera, as alarm compounds [55, 56]. Pentyl butanoate and 2-methyl hexanoic acid were identified in BGs of all *T. dimidiata* haplogroups, although neither has been reported from disturbed bugs of any other triatomine species [12]. Both *T. pallidipennis* and *T. phyllosoma* secreted these compounds when agitated, but they were not secreted by disturbed *T. longipennis*, even though the latter species produces the compounds in BGs. Propanoic acid is a repellent for grain storage beetles and weevils, is secreted by Coleoptera, some aphids, and has also been reported from disturbed adult *R. prolixus* and *T. infestans* [47, 48, 56].

Behaviourally, disturbed *T. pallidipennis* and *T. phyllosoma* emitted similar compounds, both dissimilar to those from *T. longipennis.* Additionally, this latter species emitted twice as many MG compounds when disturbed (17), compared to either of the former. Although phenetic analyses have until now reported minimal differences among species of the *phyllosoma* complex, despite phylogenetic inferences using ITS2, *cytb*, and RAPD [25, 57, 58], this was assumed to be due to recent divergence (1–10 my) [59–62]. Similar to morphometric differences which are expected when populations select for specific behaviours and micro-environmental conditions (host nest types, geography and climate), the degree and nature of landscape modification and fragmentation patterns may also be drivers of population diversification. Analysis of VOCs in other species of the *phyllosoma* complex, and the remaining North American species complexes (*protracta*, *rubida* and *lecticularia*), will undoubtedly provide a better understanding of the phylogenetic relationships and variability between and within North American triatomines, and macro- as well as micro-environmental determinants of their distributions.

## Conclusions

Discriminant analysis of volatile compounds from disturbed bugs, and Brindley’s and metasternal glands indicate significant differentiation among the three species of the *phyllosoma* complex. The two rose oxide isomers identified in females of all three species are novel and are the first monoterpenes with a tetrahydropyrane ring reported from the Triatominae. These compounds are not found in species of the *dimidiata* complex, which share a common ancestor. Behaviourally, disturbed *T. pallidipennis* and *T. phyllosoma* (allopatric) emitted similar compounds, while both were dissimilar to those from *T. longipennis* (sympatric with *T. pallidipennis*). VOCs have provided additional evidence to discriminate species within the *phyllosoma* complex, and between the former and those from the *dimidiata* complex, despite their recent divergence. Similar analyses of the remaining species of the *phyllosoma* complex and the other North American species complexes, will provide a more complete understanding of the phylogenetic relationships at species and complex levels.

## Abbreviations

BG: Brindley’s glands
CRISP/INSP: Centro Regional de Investigación en Salud Pública/Instituto Nacional de Salud Pública
GC: Gas chromatograph
GC-MS: Gas chromatograph-mass spectrometer
ITS: Internal transcribed spacer
MG: Metasternal glands
MS: Mass spectral
NZW rabbit: New Zealand White rabbit
PDMS-DVB: Polydimethylsiloxane-divinylbenzene
RAPD: Rapid amplification of polymorphic DNA
RH: Relative humidity
SPME: Solid-phase microextraction
VOC: Volatile organic compound

## References

[1] Steiger S, Schmitt T, Schaefer HM (2001). The origin and dynamic evolution of chemical information transfer. Proc R Soc B Biol Sci.

[2] Percy-Cunningham JE, MacDonald JA, Prestwich GD, Blomquist GJ (1987). Biology and ultrastructure of sex pheromone-producing glands. Pheromone biochemistry.

[3] Davis TS, Crippen TL, Hofstetter RW, Tomberlin JK (2013). Microbial volatile emissions as insect semiochemicals. J Chem Ecol.

[4] Bickford D, Lohman DJ, Sodhi NV, Ng PKL, Meier R, Winker K (2007). Cryptic species as a window on diversity and conservation. Trends Ecol Evol.

[5] Symonds MR, Elgar MA (2008). The evolution of pheromone diversity. Trends Ecol Evol.

[6] Oldham NJ. Chemical studies on exocrine gland secretions and pheromones of some social insects. PhD Thesis, Keele University, UK; 1994. pp. 201.

[7] Seybold SJ, Quilici DR, Tillman JA, Vanderwel D, Wood DL, Blomquist GJ. De novo biosynthesis of the aggregation pheromone components ipsenol and ipsdienol by the pine bark beetles *Ips paraconfusus* Lanier and *Ips pini* (Say) (Coleoptera: Scolytidae). Proc Natl Acad Sci USA. 1995;92:8393–7. 10.1073/pnas.92.18.8393 PMC41163 11607576

[8] Kälin M, Barrett FM (1975). Observations on the anatomy, histology, reléase site, and function of Brindley’s glands in the blood-sucking bug, *Rhodnius prolixus* (Heteroptera: Reduviidae). Ann Entomol Soc Am.

[9] Cruz-López L, Malo EA, Rojas JC, Morgan ED (2001). Chemical ecology of triatomine bugs: vectors of Chagas disease. Med Vet Entomol.

[10] Manrique G, Vitta AC, Ferreira RA, Zani CL, Unelius CR, Lazzari CR (2006). Chemical communication in Chagas disease vectors. Source, identity and potential function of volatiles released by the metasternal and Brindley's glands of *Triatoma infestans* adults. J Chem Ecol.

[11] May-Concha I, Rojas JC, Cruz-López L, Millar JG, Ramsey JM (2013). Volatile compounds emitted by *Triatoma dimidiata*, a vector of Chagas disease: chemical identification and behavioural analysis. Med Vet Entomol.

[12] May-Concha IJ, Rojas-Leon JC, Cruz-López L, Ibarra-Cerdeña CN, Ramsey JM (2015). Volatile compound diversity and conserved alarm behaviour in *Triatoma dimidiata*. Parasit Vectors.

[13] Ibarra-Cerdeña CN, Zaldivar-Riveron A, Peterson AT, Sanchez-Cordero V, Ramsey JM. Phylogeny and niche conservatism in North and Central American triatomine bugs (Hemiptera: Reduviidae: Triatominae), vectors of Chagas’ disease. PLoS Negl Trop Dis. 2014;8:e3266. 10.1371/journal.pntd.0003266 PMC4214621 25356550

[14] Pontes GB, Bohman B, Unelius CR, Lorenzo MG (2008). Metasternal gland volatiles and sexual communication in the triatomine bug, *Rhodnius prolixus*. J Chem Ecol.

[15] Vitta RAC, Bohman B, Unelius CR, Lorenzo MG (2009). Behavioral and electrophysiological responses of *Triatoma brasiliensis* males to volatiles produced in the metasternal glands of females. J Chem Ecol.

[16] Lent H, Wygodzinsky P (1979). Revision of the Triatominae (Hemiptera, Reduviidae), and their significance as vector of Chagas’ disease. Bull Am Mus Nat Hist.

[17] Zárate LG, Zárate RJ (1985). A checklist of the Triatominae (Hemiptera: Reduviidae) of México. Intl J Entomol.

[18] Ibarra-Cerdeña CN, Sánchez-Cordero V, Peterson AT, Ramsey JM. Ecology of North American Triatominae. Acta Trop. 2009;110:178–86. 10.1016/j.actatropica.2008.11.012 19084490

[19] Ramsey JM, Peterson AT, Carmona-Castro O, Moo-Llanes DA, Nakazawa Y, Butrick M (2015). Atlas of Mexican Triatominae (Reduviidae: Hemiptera) and vector transmission of Chagas disease. Mem Ins Oswaldo Cruz.

[20] Magallón E, Lozano F, Flores A, Bosseno MF, Brenière SF. Sylvatic Triatominae of the *phyllosoma* complex (Hemiptera: Reduviidae) around the community of Carrillo Puerto, Nayarit, Mexico. J Med Entomol. 2001;38:638–40. 10.1603/0022-2585-38.5.638 11580035

[21] Ramsey JM, Gutierrez-Cabrera AE, Salgado-Ramírez K, Peterson AT, Sánchez-Cordero V, Ibarra-Cerdeña CN (2012). Ecological connectivity of *Trypanosoma cruzi* reservoirs and *Triatoma pallidipennis* host in an anthropogenic landscape with endemic Chagas disease. PLoS One.

[22] Martínez-Tovar JG, Rodríguez-Rojas JJ, Arque-Chunga W, Lozano-Rendón JA, Ibarra-Juárez LA, Dávila-Barbosa JA (2013). Nuevos registros geográficos y notas de infección de *Triatoma gerstaeckeri* (Stål) y *Triatoma rubida* (Uhler) (Hemiptera: Reduviidae: Triatominae) en Nuevo León y Coahuila. México Acta Zool Mex.

[23] López-Cancino SA, Tun-Ku E, De la Cruz-Felix HK, Ibarra-Cerdeña CN, Izeta-Alberdi A, Pech-May A, et al. Landscape ecology of *Trypanosoma cruzi* in the southern Yucatan Peninsula. Acta Trop. 2015;151:58–72. 10.1016/j.actatropica.2015.07.021 26219998

[24] Ibarra-Cerdeña CN, Valiente-Banuet L, Sánchez-Cordero V, Stephens CR, Ramsey JM (2017). *Trypanosoma cruzi* reservoir-triatomine vector co-occurrence networks reveal meta-community effects by synanthropic mammals on geographic dispersal. PeerJ.

[25] Espinoza B, Martínez-Ibarra JA, Villalobos G, De la Torre P, Laclette JP, Martínez-Hernández F. Genetic variation of North American triatomines (Insecta: Hemiptera: Reduviidae): initial divergence between species and populations of Chagas disease vector. Am J Trop Hyg. 2013;88:275–84. 10.4269/ajtmh.2012.12-0105 PMC3583317 23249692

[26] Martínez-Hernández F, Martínez-Ibarra JA, Villalobos G, De la Torre P, Laclette JP, Alejandré-Aguilar R (2010). Natural crossbreeding between sympatric species of the *phyllosoma* complex (Insecta: Hemiptera: Reduviidae) indicate the existence of one species with morphologic and genetic variations. Am J Trop Med Hyg.

[27] Martinez-Ibarra JA, Noqueda-Torres B, Licón-Trillo A, Alejandre-Aguilar R, Salazar-Schettino PM, Vences-Blanco MO (2015). Biological aspects of crosses between *Triatoma recurva* (Stål), 1868 (Hemiptera: Reduviidae: Triatominae) and other members of the *Phyllosoma* complex. J Vector Ecol.

[28] Valdez-Tah AR, Huicochea-Gómez L, Nazar-Beutelspacher A, Ortega-Canto J, Ramsey JM (2015). La vulnerabilidad humana a la transmisión vectorial de *Tripanosoma cruzi* a través de los procesos de salud-enfermedad y la apropiación social del territorio. Salud Colectiva.

[29] Pavia DL, Lampman GM, Kriz GS, Engel RG (1990). Introduction to organic laboratory techniques, a microscale approach.

[30] Dujardin JP Site: http://www.mpl.ird.fr/morphometrics/clic/index.html. 2004.

[31] May-Concha I, Guerenstein P, Ramsey JM, Rojas JC, Catalá S. Antennal phenotype of Mexican haplogroups of the *Triatoma dimidiata* complex, vector of Chagas disease. Infect Genet Evol. 2016;40:73–9. 10.1016/j.meegid.2016.02.027 26921798

[32] Marcilla A, Bargues MD, Ramsey JM, Magallon-Gastelum E, Salazar-Schettinom PM, Abad-Franch F (2001). The ITS-2 of the nuclear rDNA as a molecular marker for populations, species, and phylogenetic relationships in Triatominae (Hemiptera: Reduviidae), vectors of Chagas disease. Mol Phylogenet Evol.

[33] Rossiter M, Staddon BW (1983). 3-Methyl-2-hexanone from the bug *Dipetalogaster maximus* (Uhler) (Heteroptera; Reduviidae). Experientia.

[34] Hypša V, Tietz DF, Zrzavý J, Rego ROM, Galvao C, Jurberg J (2002). Phylogeny and biogeography of Triatominae (Hemiptera: Reduviidae): molecular evidence of a new world origin of the Asiatic clade. Mol Phylogenet Evol.

[35] Ohloff G, Ohloff G, Kölbel H, Kurzendörfer P, Sackmann H, Demus D (1969). Chemie der Geruchs- und Geschmacksstoffe. Angewandte Chemie. Fortschritte der Chemischen Forschung.

[36] von Sydow E, Karlsson G. The aroma of black currants. IV. The influence of heat measured by instrumental methods. Lebensmittel-Wiss. u. Technol. 1971;4:54–8.

[37] Nishimura K, Sakai T, Ogawa M, Hirose Y (1964). Analysis of the volatile constituents of hamanasu absolute oil. Bull Chem Soc Jap.

[38] Yilmaztekin M (2014). Analysis of volatile components of cape gooseberry (*Physalis peruviana* L.) grown in Turkey by HS-SPME and GC-MS. Sci World J.

[39] Vidari G, De Bernardi M, Pavan M, Ragozzino L. Rose oxide and iridiodal from *Aromia moschata* L. (Coleoptera: Cerambicidae). Tetrahedron Lett. 1973;41:4065–8.

[40] Moore BP, Brown WV (1976). The chemistry of the metasternal gland secretion of the eucalypt longicorn, *Phoracantha synonyma* (Coleoptera: Cerambycidae). Aust J Chem.

[41] Ohmura W, Hishiyama S, Nakashima T, Kato A, Makihara H, Ohira T, et al. Chemical composition of the defensive secretion of the longhorned beetle, *Chloridolum loochooanum*. J Chem Ecol. 2009;35:250–5. 10.1007/s10886-009-9591-y 19159979

[42] Cruz-López L, Malo AE, Morgan DE, Rincon M, Guzman M, Rojas JC (2005). Mandibular gland secretion of *Melipona beecheii*: chemistry and behavior. J Chem Ecol.

[43] Dettner K (1987). Chemosystematics and evolution of beetle chemical defenses. Annu Rev Entomol.

[44] Seybold SJ (1993). Role of chirality in olfactory-directed behaviour: aggregation of pine engraver beetles in the genius *Ips* (Coleoptera: Scolytidae). J Chem Ecol.

[45] Blum MS (1981). Chemical defenses of arthropods.

[46] Juárez P, Brenner R (1981). Bioquimica del ciclo evolutivo de *Triatoma infestans* (vinchuca). V. Emisión de ácidos grasos volátiles. Acta Fisiol Latinoamericana.

[47] Cruz-López L, Morgan ED, Ondarza RN (1995). Brindley’s gland exocrine products of *Triatoma infestans*. Med Vet Entomol.

[48] Guerenstein PG, Guerin PM (2004). A comparison of volatiles emitted by adults of three triatomine species. Entomol Exp Appl.

[49] Apps PJ, Viljoen HW, Pretorius V (1988). Aggregation pheromones of the bont tick *Amblyomma hebraeum*: identification of candidates for bioassay. Onderstepoort J Vet Res.

[50] Schofield CJ (1979). The behaviour of Triatominae (Hemiptera: Reduviidae): a review. Bull Entomol Res.

[51] Ward JP (1981). A comparison of the behavioural responses of the haematophagous bug, *Triatoma infestans* to synthetic homologues of two naturally occurring chemicals (n- and isobutyric acid). Physiol Entomol.

[52] Minoli S, Palottini F, Crespo JG, Manrique G (2013). The main component of an alarm pheromone of kissing bugs plays multiple roles in the cognitive modulation of the escape response. Front Behav Neurosci.

[53] Palottini F, Manrique G (2016). Compounds released by disturbed adults of the haematophagous bug *Triatoma infestans* (Hemiptera: Reduviidae): behavioural effects of single compounds and binary mixtures. Physiol Entomol.

[54] Rojas JC, Rios-Candelaria E, Cruz-Lopez L, Santiesteban A, Bond-Compean JG, Brindis Y (2002). A reinvestigation of Brindley’s gland exocrine compound of *Rhodnius prolixus* (Hemiptera: Reduviidae). J Med Entomol.

[55] González-Audino GP, Alzogaray RA, Vannesa C, Masuh H, Fontán A, Gatti P (2006). Volatile compounds secreted by Brindley's glands of adult *Triatoma infestans*: identification and biological activity of previously unidentified compounds. J Vect Ecol.

[56] Germinara GS, De Cristofaro A, Rotundo G (2008). Behavioral responses of adult *Sitophilus granarius* to individual cereal volatiles. J Chem Ecol.

[57] Brenière SF, Taveira B, Bosseno MF, Ordoñez R, Lozano-Kasten F, Magallon-Gastellum E (2003). Preliminary results of random amplification of polymorphic DNA among Triatominae of the *phyllosoma* complex (Hemiptera, Reduviidae). Mem Inst Oswaldo Cruz.

[58] Martínez HF, Villalobos CG, Ceballos AM, De la Torre P, Laclette JP, Alejandre-Aguilar R (2006). Phylogenetic analysis of Triatominae (Hemiptera: Reduviidae) species of epidemiological importance in the transmission of Chagas disease: nuclear DNA *vs* mitochondrial DNA as molecular markers. Mol Phylogenet Evol.

[59] Juárez MP, Blomquist GJ (1993). Cuticular hydrocarbons of *Triatoma infestans* and *T. mazzottii*. Comp Biochem Physiol.

[60] Juárez MP, Carlson DA, Salazar-Schettino PM, Mijailovski S, Rojas G (2002). Cuticular hydrocarbons of Chagas disease vectors in Mexico. Mem Inst Oswaldo Cruz.

[61] Catalá S, Sachetto C, Moreno M, Rosales R, Salazar-Schettino PM, Gorla D (2005). Antennal phenotype of *Triatoma dimidiata* populations and its relationship with species of *phyllosoma* and *protracta* complexes. J Med Entomol.

[62] Calderón-Fernández GM, Juárez P (2013). Cuticular hydrocarbons of the *Triatoma sordida* species subcomplex (Hemiptera: Reduviidae). Mem Inst Oswaldo Cruz.

